# Two Distinct Groups Are Shown to Be at Risk of Diabetes by Means of a Cluster Analysis of Four Variables

**DOI:** 10.3390/jcm12030810

**Published:** 2023-01-19

**Authors:** Ryoma Ito, Satoru Mizushiri, Yuki Nishiya, Shoma Ono, Ayumi Tamura, Kiho Hamaura, Akihide Terada, Jutaro Tanabe, Miyuki Yanagimachi, Kyi Mar Wai, Yutaro Kudo, Kazushige Ihara, Yoshiko Takahashi, Makoto Daimon

**Affiliations:** 1 Department of Endocrinology and Metabolism, Hirosaki University Graduate School of Medicine, Hirosaki 036-8562, Japan; 2 Department of Social Medicine, Hirosaki University Graduate School of Medicine, Hirosaki 036-8562, Japan; 3 Center of Innovation Research Initiatives Organization, Hirosaki 036-8562, Japan

**Keywords:** cluster analysis, incident type 2 diabetes, nondiabetic population

## Abstract

Recent attempts to classify adult-onset diabetes using only six diabetes-related variables (GAD antibody, age at diagnosis, BMI, HbA1c, and homeostatic model assessment 2 estimates of b-cell function and insulin resistance (HOMA2-B and HOMA2-IR)) showed that diabetes can be classified into five clusters, of which four correspond to type 2 diabetes (T2DM). Here, we classified nondiabetic individuals to identify risk clusters for incident T2DM to facilitate the refinement of prevention strategies. Of the 1167 participants in the population-based Iwaki Health Promotion Project in 2014 (baseline), 868 nondiabetic individuals who attended at least once during 2015–2019 were included in a prospective study. A hierarchical cluster analysis was performed using four variables (BMI, HbA1c, and HOMA2 indices). Of the four clusters identified, cluster 1 (n = 103), labeled as “obese insulin resistant with sufficient compensatory insulin secretion”, and cluster 2 (n = 136), labeled as “low insulin secretion”, were found to be at risk of diabetes during the 5-year follow-up period: the multiple factor-adjusted HRs for clusters 1 and 2 were 14.7 and 53.1, respectively. Further, individuals in clusters 1and 2 could be accurately identified: the area under the ROC curves for clusters 1and 2 were 0.997 and 0.983, respectively. The risk of diabetes could be better assessed on the basis of the cluster that an individual belongs to.

## 1. Introduction

Type 2 diabetes (T2DM) increases the risks of serious physical and mental health problems, and its prevalence is increasing worldwide [1,2]. Therefore, the identification of individuals at risk of developing T2DM is important. To this end, biological markers may be of use in clinical settings, regardless of whether they show causal relationships or merely an association with T2DM. However, currently, no effective markers other than those directly related to glucose metabolism, such as glucose concentrations and glycated substrates, are used in clinical practice [3]. Although such markers are an undoubtedly effective means of predicting incident diabetes, they are not useful in assessing the T2DM status classification, which will occur in the future.

Since T2DM is a heterogeneous disorder of the glucose metabolism that is characterized by both insulin resistance and pancreatic β-cell dysfunction [4,5], the pathophysiology associated with the development of T2DM and the clinical characteristics of individuals with T2DM vary substantially from person to person. To evaluate such differences in the underlying condition in individuals with diabetes, a cluster analysis of six variables (glutamate decarboxylase antibody (GAD-Ab), age at diagnosis, HbA1c, BMI, and homeostatic model assessment 2 estimates of b-cell function and insulin resistance (HOMA2-B and HOMA2-IR)) has recently been conducted, and this identified five clusters with distinct clinical characteristics and outcomes, such as diabetic complications [6]. Of these five clusters, one seemed to correspond to type 1 diabetes, and the other four seemed to correspond to T2DM. Since then, various other studies have similarly shown that T2DM can be classified at least into four groups, labeled as severe insulin-deficient diabetes (SIDD), severe insulin-resistant diabetes (SIRD), mild obesity-related diabetes (MOD), and mild age-related diabetes (MARD) [6,7,8,9,10,11,12,13,14,15,16,17,18]. In addition, individuals in these T2DM clusters have been shown to be at different risks of diabetic complications and to have differences in their glycemic response, with particular benefits of certain antidiabetic drugs for particular clusters [6,8,9,10,11,12,16,17,18]. Thus, T2DM can be classified into four groups, which necessitate differing therapeutic approaches. However, it is unclear whether nondiabetic individuals could be similarly classified. If so, individuals at risk of diabetes could be evaluated more precisely with respect to their underlying pathophysiology, which should help suggest the most appropriate means of preventing T2DM.

Therefore, in the present study, we classified nondiabetic individuals in the general Japanese population by hierarchical clustering analyses using four variables: HbA1c, BMI, HOMA2-B, and HOMA2-IR. Furthermore, we evaluated the risk of incident diabetes in each group during a 5-year follow-up period, along with the factors related to the development of diabetes in each cluster. The findings of the present study suggest a means of precisely evaluating individuals at risk of diabetes, permitting the targeted provision of healthcare services for the prevention of T2DM.

## 2. Methods

### 2.1. Study Sample

Participants were recruited from the residents aged ≥20 years living in the Iwaki area, Japan, through a public announcement (the Iwaki Health Promotion Project: Iwaki study). The Iwaki study is aimed to prevent lifestyle-related diseases and prolong lifespans, and no inclusion and exclusion criteria were set [19,20,21]. The Iwaki study is conducted annually in the Iwaki area of the city of Hirosaki in Aomori Prefecture, Northern Japan. Of the 1167 individuals who participated in the Iwaki study in 2014, 979 individuals attended the follow-up examinations at various intervals until 2019. Of these participants, 19 with an incomplete dataset and 74 with diabetes were excluded. After these exclusions, 886 individuals (327 men and 559 women) aged 53.8 ± 14.6 years remained for inclusion in the present study. The mean duration of follow-up was 4.9 years.

This study was approved by the Ethics Committee of the Hirosaki University School of Medicine (No. 2014-014, 2014-377-1, 2016-028-1, 2021-030, 2018-063, and 2019-009) and was conducted in accordance with the principles of the Declaration of Helsinki. Written informed consent was obtained from all the participants.

### 2.2. Parameters Measured

Blood samples were collected in the morning from a peripheral vein of fasted participants. Urine samples were also collected in the morning. The following parameters were measured: height; body weight; BMI; percentage body fat (fat (%)); fasting blood glucose (FBG); fasting serum C-peptide; glycated hemoglobin (HbA1c); systolic and diastolic blood pressure; serum low-density lipoprotein (LDL)-cholesterol, triglyceride (TG), high-density lipoprotein (HDL)-cholesterol, uric acid, urea nitrogen, and creatinine concentrations; and urinary albumin and creatinine concentrations (uACR). Fat (%) was measured using the bioelectricity impedance method with a Tanita MC-190 body composition analyzer (Tanita Corp., Tokyo, Japan). HbA1c (%) is expressed as the National Glycohemoglobin Standardization Program value. Laboratory testing was performed in a commercial laboratory (LSI Medience Co., Tokyo, Japan), according to the reagent manufacturer’s protocols. Pancreatic *β*-cell function (B) and insulin resistance (IR) were evaluated using the updated Homeostatic model assessment (HOMA2) via the HOMA 2 Calculator (©Oxford University 2004 (available at www.dtu.ox.ac.uk/homacalculator/download.php) by inserting the serum C-peptide and blood glucose concentrations. The incidence and/or treatment of diabetes was identified using a questionnaire. DM was defined based on the criteria of the Japan Diabetes Society published in 2010: FBG ≥ 126 mg/dL [22]. In participants in whom the FBG concentration was not measured, diabetes was defined using an HbA1c of ≥6.5%. Participants receiving treatment for diabetes were also considered to have diabetes. Hypertension was defined using a blood pressure of ≥140/90 mmHg or current treatment for hypertension. Hyperlipidemia was defined using an LDL-cholesterol of ≥140 mg/dL, a TG of ≥150 mg/dL, or current treatment for hyperlipidemia. Alcohol consumption (current or not) and smoking (never, past, or current smoker) habits were recorded using questionnaires.

### 2.3. Statistical Analysis

Data are presented as means ± SDs. Statistical significance of differences in values among groups (parametric) and case–control associations among groups (nonparametric) were assessed using the analysis of covariance (ANOVA) with Tukey’s post-hoc analyses and the χ2 test, respectively. Statistical significance of differences in parametric and nonparametric values for participants in each cluster at baseline and at the onset of diabetes were assessed using the paired *t*-test and McNemar’s test, respectively. Based on their HbA1c, BMI, HOMA2-B, and HOMA2-IR, the participants were grouped by hierarchical clustering into four major clusters, and the risk of diabetes in each cluster was evaluated using Kaplan–Meier and multivariate Cox proportional hazard regression analyses. Cox proportional hazard regression models were used to calculate hazard ratios (HRs) for incident diabetes at baseline after adjustment for multiple possible confounding factors, including age and gender. Receiver operating characteristic (ROC) curves were plotted to determine the optimal cut-off values for each cluster at baseline. The values that yielded the highest sensitivities and specificities were determined as the cut-off values. Prior to statistical analysis, HOMA-IR, TG concentrations, and uACR were log-transformed (log10) to approximate a normal distribution. *p* < 0.05 was considered to represent statistical significance. All analyses were performed using JMP version 16.0 (SAS Institute Japan Ltd., Tokyo, Japan).

## 3. Results

### 3.1. Clinical Characteristics of the Participants at the Baseline

The clinical characteristics of the participants at baseline are shown in Table 1. The mean age of the participants was 53.8 ± 14.6. Although the national prevalence of hypertension and hyperlipidemia for nondiabetic individuals has not been reported, the prevalence of hypertension (42.1%) and hyperlipidemia (36.5%) measured appeared to not be substantially different from those of the general Japanese population: the national prevalence of hypertension reported by the Japanese government in 2014 were 36.2% and 26.8% for men and women of ≥20 years of age, respectively; and the prevalence of hyperlipidemia reported in other areas of Japan was also similar [23,24,25,26].

### 3.2. Cluster Analysis Using Four Variables

**Figure 1:**
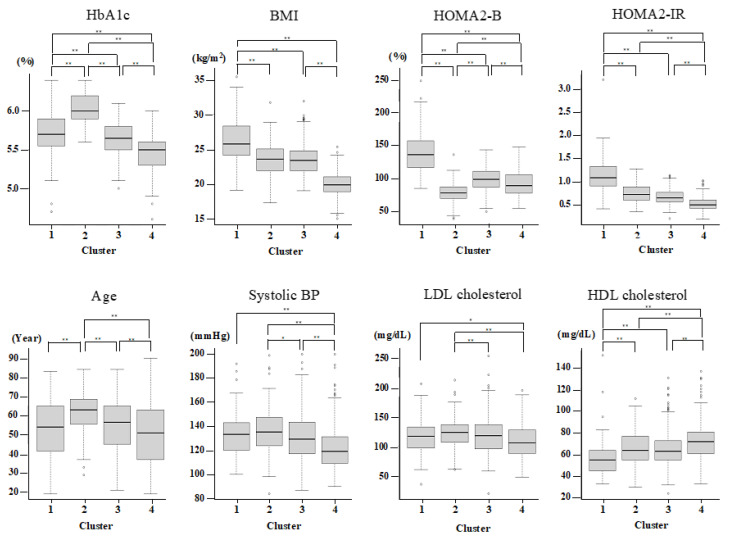
Cluster characteristics. The characteristics of HbA1c, BMI, HOMA2-IR, HOMA2-B, Age, sBP, LDL, and HDL-cholesterol for each cluster. *p* < 0.05 and <0.01 are indicated by * and **, respectively.

Since only nondiabetic individuals were included, two variables (glutamate decarboxylase antibody and age at diagnosis) out of the six original variables reported previously were not used for the present cluster analysis. Therefore, on the basis of HbA1c, BMI, HOMA2-B, and HOMA2-IR, the participants were grouped by hierarchical clustering into four major clusters (Table 2 and Figure 1). Cluster 1 (n = 103 (11.6%)) was characterized by high BMI, insulin resistance, high insulin secretion, an abnormal lipid profile, and higher kidney damage and was labeled as “obese insulin resistant with sufficient compensatory insulin secretion” (IR-SIS). Cluster 2 (n = 136 (15.3%)) was characterized by low insulin secretion and dysglycemia and was labeled as “low insulin secretion” (Low-IS). Cluster 3 (n = 314 (35.4%)) was characterized by normal BMI and a generally healthy metabolic profile and was labeled as “non-obese healthy”. Cluster 4 (n = 333 (37.6%)) was characterized by a low BMI and a generally healthy metabolic profile and was labelled as “lean healthy”.

### 3.3. Risk of Diabetes Associated with Clusters

**Figure 2:**
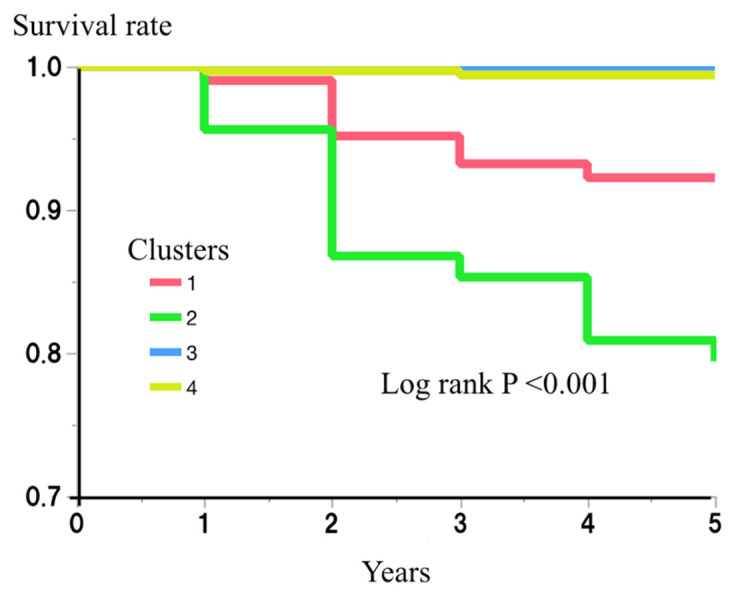
Risk of clusters for incident diabetes.

The risk of incident diabetes for each cluster was then examined. During the 5-year follow-up period of the study, 38 (4.3%) of the participants developed diabetes. The numbers who developed diabetes during this period were 8/103 (7.8%), 28/136 (20.6%), 0/314 (0.0%), and 2/333 (0.6%), for clusters 1–4, respectively. An analysis using the Kaplan–Meier method showed a significantly higher risk of diabetes in clusters 1 and 2 (log rank *p* < 0.001) (Figure 2). Cox’s proportional hazard regression model analysis also showed the effects of being in clusters 1 and 2 on the risk of incident diabetes (hazard ratio (HR) (95% confidence interval (CI)) vs. being in clusters 3 or 4: 25.8 (5.5–121.6) and 72.0 (17.2–302.3), respectively) (Table 3). After further adjustment for multiple possible confounding factors (age, gender, sBP, LDL-c, HDL-c, SUN, Cre, SUA, and alcohol drinking and smoking habits), being in clusters 1 and 2 remained a risk factor for incident diabetes (HR and 95% CI: 14.2 (2.9–70.4) and 53.2 (12.4–227.5), respectively) (Table 3).

The risk of each cluster for incident diabetes during the 5-year follow-up period was then examined using the Kaplan–Meier method. The differences among the clusters were assessed using log-rank test. *p* < 0.05 was considered to represent statistical significance.

### 3.4. Cut-Off Values of the Four Variables for the Identification of Participants Belonging to Clusters 1 and 2

**Figure 3:**
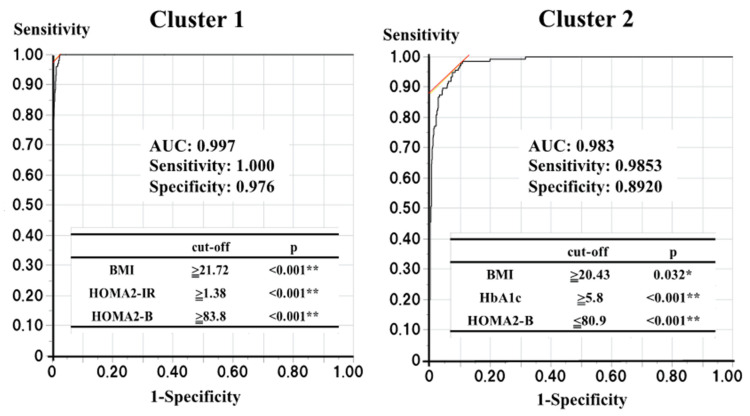
The optimal cut-off values to find the subjects belonging to clusters 1 and 2. *p* < 0.05 and <0.01 are indicated by * and **, respectively.

Next, to identify participants belonging to clusters 1 and 2 or at risk of diabetes in a standard clinical setting, we determined the optimal cut-off values of the four variables used for the cluster analysis. Logistic regression analyses showed that someone in cluster 1 could be identified using three of the four variables (the *p*-values for HbA1c, BMI, HOMA2-B, and HOMA2-IR were 0.104, <0.001, <0.001, and <0.001, respectively), while someone in cluster 2 could also be identified using three of the four variables (*p*-values for HbA1c, BMI, HOMA2-B, and HOMA2-IR were <0.001, 0.041, <0.001, and 0.856, respectively). Therefore, ROC curve analyses using the corresponding variables were performed to determine the optimal cut-off values for the identification of individuals belonging to clusters 1 and 2 (Figure 3). For cluster 1, BMI ≥ 21.72 kg/m^2^, HOMA2-B ≥83.8, and HOMA2-IR ≥1.38 were found to be the optimal cut-off values (area under the ROC curve (AUC): 0.997, sensitivity: 1.000, and specificity: 0.976), and for cluster 2, HbA1c >5.8%, BMI ≥20.43 kg/m^2^, and HOMA2-B ≤80.9 were found to be the optimal cut-off values (AUC: 0.983, sensitivity: 0.985, specificity: 0.892).

ROC curve analyses with those each corresponding three variables were performed to determine the optimal cut-off values for the finding the subjects belonging to the clusters 1 and 2.

### 3.5. Changes in the Clinical Characteristics of the Participants in Clusters 1 and 2 between Baseline and the Onset of Diabetes

Since clinical characteristics of the participants in clusters 1 and 2 at the baseline were very different, the factors predisposed toward the development of diabetes in each cluster may be different. Therefore, we next evaluated the changes in the clinical characteristics in the participants in clusters 1 and 2 between the baseline and the onset of diabetes (Table 4). The participants in cluster 1 who developed diabetes showed a decline of compensatory increased insulin secretion (from 129.9 ± 26.4 to 99.2 ± 20.3, *p* = 0.029), without a concomitant decrease in insulin resistance (from 1.47 ± 0.39 to 1.84 ± 0.98, *p* = 0.340) during the follow-up period, while the participants in cluster 2 who developed diabetes showed a significant increase in insulin resistance (from 0.84 ± 0.20 to 1.18 ± 0.49, *p* < 0.001), along with a modest decrease in insulin secretion (from 72.9 ± 17.4 to 64.6 ± 18.8, *p* = 0.005).

## 4. Discussion

In the present study of nondiabetic Japanese participants, we identified two distinct groups who are at risk of incident diabetes using a cluster analysis of four variables (HbA1c, BMI, HOMA2-B, and HOMA2-IR). Thus, in addition to T2DM being a heterogenous disease, we have shown that those at risk of diabetes are also heterogenous in their characteristics. Of the two groups found to be at risk of diabetes, participants in cluster 1 (IR-SIS) were not dysglycemic and therefore would not be identified to be at risk of incident diabetes over the relatively short period of 5 years in a standard clinical setting. In contrast, the participants in cluster 2, although not diabetic, showed dysglycemia and therefore would be more readily assessed as being at risk of diabetes. Dysglycemia is a well-known marker of diabetes risk. However, although the risk of diabetes associated with dysglycemia, defined as HbA1c 5.8~6.4%, was very high (HR (95% CI): 12.2 (4.3–35.3), *p* < 0.001), it appeared to be lower than that for cluster 2 (HR (95% CI): 53.1 (12.4–227.5), *p* < 0.001), suggesting that being in cluster 2 is a superior predictor of diabetes than dysglycemia alone. In addition, the ROC analysis showed that the two groups could be evaluated highly reliably using only the cut-off values for the three corresponding variables (AUCs of 0.9970 and 0.9831 for clusters 1 and 2, respectively). Thus, using only four commonly measured variables, two distinct groups at risk of diabetes within the following 5 years can be accurately identified, at least in the Japanese population.

Moreover, because individuals at risk of incident diabetes can be divided into two groups with differing clinical characteristics and predisposing factors, healthcare can be tailored to individuals according to the group to which they belong. In other words, individuals in clusters 1 or 2 should both be eligible for more intensive healthcare, but the specific measures instituted should be determined by the cluster to which they belong.

As described, cluster 1 was characterized by high BMI, insulin resistance, high insulin secretion, an abnormal lipid profile, and higher kidney damage, and it was labeled as “obese insulin resistant with sufficient compensatory insulin secretion” (IR-SIS). Bearing the facts in mind, the participants in cluster 1 who developed diabetes showed declines in the compensatory increased insulin secretion (from 129.9 ± 26.4 to 99.2 ± 20.3, *p* = 0.029), without a concomitant decrease in insulin resistance (change from 1.47 ± 0.39 to 1.84 ± 0.98, *p* = 0.340) during the 5-year follow-up period. Interestingly, the ROC analysis, which was used to determine the cut-off values for each of the variables defining cluster 1, revealed that individuals with high insulin secretion (HOMA2-B ≥83.8), moderate-to-high BMI (≥21.72), and high insulin resistance (HOMA2-IR ≥1.3831) can be allocated to cluster 1. In other words, higher, rather than lower, insulin secretion is a predictor of being in cluster 1. These findings suggest that excessive insulin secretion in compensation for insulin resistance may be followed by a rapid decline in insulin secretion. A recent cluster analysis using six variables (glutamate decarboxylase antibody, age at diagnosis, HbA1c, BMI, HOMA2-B, and HOMA2-IR) showed four T2DM clusters with distinctly different clinical characteristics and outcomes, such as diabetic complications [6,7,8,9,10,11,12,13,14,15,16,17,18]. If the characteristics of cluster 1 at the onset of diabetes are compared with those of the four T2DM clusters previously described, cluster 1 appears to be similar to MOD, which has been shown to be associated with the lowest risk of diabetic complications of the four defined clusters [6,8,9,10,11,12,16,17,18]. This implies that individuals in cluster 1 are at risk of incident diabetes, but if they do develop diabetes, their risks of developing diabetes-related complications may be low. Taken together, these findings suggest that the healthcare for individuals in cluster 1 should focus on obesity reduction, with the aim of reducing insulin resistance and avoiding the decline in compensatory increased insulin secretion.

Cluster 2 was characterized by low insulin secretion and dysglycemia and was labeled as “low insulin secretion” (Low-IS). The participants in cluster 2 who developed diabetes showed significant increases in insulin resistance (from 0.84 ± 0.20 to 1.18 ± 0.49, *p* < 0.001), along with a modest decrease in insulin secretion (from 72.9 ± 17.4 to 64.6 ± 18.8, *p* = 0.005) during the 5-year follow-up period. However, ROC curve analyses to define optimal cut-off values for the variables predicting cluster 2 revealed that HbA1c >5.8, BMI ≥20.43, and HOMA2-B ≤80.9 were the values. Namely, insulin resistance did not predict being in cluster 2. Taken together, these findings suggest that a slow decline in insulin secretion over a period of 5 years, coupled with a modest increase in insulin resistance and low insulin secretion at baseline, may lead to the development of diabetes. Therefore, to maintain adequate insulin secretion, healthcare should be focused on preventing an increase in blood glucose concentrations for individuals in cluster 2. In addition, as for cluster 1, we also compared the characteristics of cluster 2 at the onset of diabetes to those of the four T2DM clusters previously reported and found that cluster 2 is similar to MARD, which has been shown to have the highest prevalence but the lowest risk of diabetic complications among the four T2DM clusters reported in most previous studies [6,8,9,10,11,12,16,17,18,27]. This implies that individuals in cluster 2 are at higher risk of incident diabetes, but their risk of subsequently developing diabetes complications may be low, at least over a 5-year period.

As described, most of the participants who developed T2DM during the 5-year follow-up period could be classified as either MOD (21.6%) or MARD (75.7%). Although ethnic differences in the frequencies of the T2DM clusters previously reported have been reported, these high frequencies of MOD and MARD are inconsistent with those reported previously [27,28]. Even in a study of a Japanese sample, the frequencies of the SIDD, SIRD, MOD, and MARD clusters were reported to be 19.0, 7.2, 28.9, and 39.5, respectively [10]. The individuals classified in the present study were those who developed T2DM during a 5-year follow-up period and therefore could only have had T2DM for a short period of time. Furthermore, a comparison of the characteristics of the participant in clusters 1 and 2 at the onset of T2DM with those of individuals defined as SIRD and SIDD suggests that they may be at least at risk of SIRD and SIDD, respectively, in the future. Taking these findings together, it seems that the participants in cluster 1 are at risk of becoming MOD in the near future and may be at risk of becoming SIRD in the distant future, whereas the participants in cluster 2 are at risk of becoming MARD in the near future and may be at risk of becoming SIDD at the distant future. This may explain the high frequencies of MOD and MARD observed in the present study. However, further analysis with a longer follow-up and more subjects is awaited to test this hypothesis.

This study was conducted in the thought that the risk of incident T2DM could be more precisely assessed by cluster analysis than by analysis with a single variable. To ensure such a thought, we also analyzed the risk of incident T2DM for BMI in the study sample, since BMI is a well-known risk factor for incident T2DM and could be a suitable example for such an analysis. BMI was found to be associated with the risk for incident T2DM (HR: 1.15 per 1 kg/m^2^, *p* = 0.007). The analysis using the optimal cutoff value for BMI (23.187) showed that the multiple factors adjusted HR for those at risk based on the BMI was 3.15 (*p* = 0.003). In addition, further adjustments with the HbA1c and HOMA2 indices made the association insignificant (HR: 1.83, *p* = 0.171). Furthermore, the multiple factors adjusted HR for those at risk based on the cutoff values of BMI 25 kg/m^2^ and 30 kg/m^2^ were 2.00 (*p* = 0.047) and 2.15 (*p* = 0.312), respectively. These values were clearly lower than those of clusters 1 and 2 observed in the present cluster analysis. Similarly, the multiple factors adjusted HR for those at risk based on the cutoff values of FBG 92 mg/dl and 110 mg/dl were 10.86 (*p* < 0.001) and 23.64 (*p* < 0.001), respectively, clearly lower than that of cluster 2 observed in the present cluster analysis. These findings may indicate that a cluster analysis with several variables such as HbA1c, BMI, and HOMA2 indices together may more accurately assess the risk of incident T2DM than each variable alone.

The present study had several strengths and limitations. With regard to its strengths, we studied a sample from the general population and accounted for multiple factors that could have confounded the statistical analyses. Furthermore, the study had a longitudinal component, as well as a cross-sectional one, which permitted us to evaluate the relationships between the clusters and incident diabetes. As for the limitations, firstly, we recruited the participants from a health promotion study rather than from among people attending health check-ups, and therefore, the participants may have been relatively health-conscious. Consistent with this, the proportion of women in the study was high (63.1%). However, in contrast, the participants may not be so healthy, since the prevalence of hypertension and hyperlipidemia seems to be somewhat higher. In addition, since the participation rate was not so high (1167/11,292: 10.3%), a selection bias may exist. However, as shown in the results, since the prevalence of hypertension and hyperlipidemia does not seem to differ substantially from the general Japanese population, the bias may not be so substantial. Secondly, we did not measure the GAD-Ab, and therefore, individuals predisposed toward type 1 diabetes may have been misevaluated. However, none of the participants developed type 1 diabetes during the 5-year follow-up period, and therefore, this is a remote possibility. In addition, the 5-year follow-up period may have been too short for this kind of longitudinal study. However, during the follow-up period, significant differences between the groups were identified, and therefore, it seemed to be sufficient for the present analysis. Thirdly, only four variables were used in the cluster analysis. Including more variables, such as family history or genetic background, would improve the sensitivity and specificity of the risk assessment for incident T2DM. In fact, a recent study using multiple variables, including genetic factors, showed that pre-diabetics could be classified into six clusters, of which the clusters with a genetically higher risk of T2DM were at a higher risk of incident diabetes than the other clusters [29]. Therefore, future analyses with more variables, such as family history or genetic background, are awaited. However, we believe the value of this study is that it assessed the risk of incident T2DM quite accurately using only four common variables.

In conclusion, we classified nondiabetic individuals selected from the general Japanese population by hierarchical clustering analyses using four variables that are commonly measured in the standard clinical setting and identified two distinct groups that are at risk of incident diabetes during the subsequent 5 years: IR-SIS and Low-IS. Furthermore, we evaluated the factors related to the development of diabetes in each cluster at risk of incident diabetes. The findings have implications for how individuals at risk of diabetes should be evaluated and how appropriately healthcare should be targeted toward individuals for the prevention of diabetes.

## Figures and Tables

**Table 1:** Clinical characteristics of the study subjects.

Characteristics	
Number (Men/Women)	886(327/559)
Age (y)	53.8 ± 14.6
Height (cm)	160.2 ± 9.1
Body weight (kg)	58.0 ± 10.8
Body mass index (kg/m^2^)	22.5 ± 3.2
Fat (%)	24.7 ± 8.0
Plasma glucose (mg/dL)	79.8 ± 9.6
C-peptide (ng/mL)	0.98 ± 0.36
HbA1c (%)	5.64 ± 0.29
HOMA2-IR	0.69 ± 0.27
HOMA2-B	96.9 ± 26.2
Systolic blood pressure (mmHg)	128.7 ± 19.2
Diastolic blood pressure (mmHg)	77.9 ± 11.0
LDL cholesterol (mg/dL)	115.5 ± 29.3
Triglyceride (mg/dL)	96.2 ± 83.3
HDL cholesterol (mg/dL)	66.3 ± 17.1
Serum uric Acid (mg/dL)	4.86 ± 1.34
Serum urea Nitrogen (mg/dL)	14.70 ± 4.19
Serum creatinin (mg/dL)	0.69 ± 0.16
uACR (mg/grCr)	23.8 ± 117.3
Hypertension: n (%)	373(42.1)
Hyperlipidemia: n (%)	323(36.5)
Drinking alcohol: n (%)	382(43.1)
Smoking (Never/Past/Current): n (%)	570/174/142(64.3/19.6/16.0)

Data are mean ± SD or number of subjects (%).

**Table 2:** Cluster characteristics.

	Cluster				
Characteristics	1 (IR-SIS)	2 (Low-IS)	3 (Non-Obese Healthy)	4 (Lean Healthy)	*p*
Number (Men/Women)	103 (58/45)	136 (50/86)	314 (127/187)	333 (92/241)	<0.001 **
Age (y)	53.0 ± 14.7 ^#2^	61.7 ± 10.5 ^#1,3,4^	54.6 ± 14.3 ^#4^	50.1 ± 15.0 ^#2,3^	<0.001 **
Height (cm)	162.5 ± 10.5 ^#2^	158.4 ± 8.7 ^#1^	160.0 ± 9.3	160.3 ± 8.4	<0.007 **
Body weight (kg)	69.42 ± 12.18 ^#2,3,4^	59.14 ± 9.75 ^#1,4^	60.74 ± 9.23 ^#1,4^	51.42 ± 7.44 ^#1,2,3^	<0.001 **
Body mass index (kg/m^2^)	26.22 ± 3.51 ^#2,3,4^	23.47 ± 2.52 ^#1,4^	23.63 ± 2.22 ^#1,4^	19.94 ± 1.79 ^#1,2,3^	<0.001 **
Fat (%)	29.10 ± 9.28 ^#2,3,4^	26.26 ± 7.46 ^#1,4^	26.68 ± 7.51 ^#1,4^	20.78 ± 6.57 ^#1,2,3^	<0.001 **
Plasma glucose (mg/dL)	79.30 ± 11.17 ^#2,3,4^	91.29 ± 10.01 ^#1,3,4^	79.14 ± 7.24 ^#2,4^	76.02 ± 6.84 ^#1,2,3^	<0.001 **
C-peptide (nmol/L)	1.64 ± 0.48 ^#2,3,4^	1.03 ± 0.25 ^#1,4^	0.97 ± 0.20 ^#1,4^	0.77 ± 0.19 ^#1,2,3^	<0.001 **
HbA1c (%)	5.71 ± 0.29 ^#2,3,4^	6.06 ± 0.18 ^#1,3,4^	5.62 ± 0.20 ^#1,2,4^	5.48 ± 0.23 ^#1,2,3^	<0.001 **
HOMA2-IR	1.15 ± 0.37 ^#2,3,4^	0.75 ± 0.19 ^#1,3,4^	0.68 ± 0.15 ^#1,2,4^	0.53 ± 0.14 ^#1,2,3^	<0.001 **
HOMA2-B	140.6 ± 32.4 ^#2,3,4^	76.9 ± 14.9 ^#1,3,4^	97.9 ± 17.2 ^#1,2,4^	90.5 ± 18.5 ^#1,2,3^	<0.001 **
Systolic blood pressure (mmHg)	133.1 ± 17.5 ^#4^	136.1 ± 18.2 ^#3,4^	131.2 ± 19.3 ^#2,4^	122.0 ± 18.0 ^#1,2,3^	<0.001 **
Diastolic blood pressure (mmHg)	82.8 ± 11.1 ^#3,4^	80.8 ± 10.7 ^#4^	78.3 ± 10.7 ^#1,4^	75.0 ± 10.6 ^#1,2,3^	<0.001 **
LDL cholesterol (mg/dL)	116.8 ± 29.0 ^#4^	123.2 ± 26.5 ^#14^	119.5 ± 30.6 ^#4^	108.0 ± 27.7 ^#1,2,3^	<0.001 **
Triglyceride (mg/dL)	168.3 ± 139.2 ^#2,3,4^	103.7 ± 107.2 ^#1,4^	97.0 ± 67.4 ^#1,4^	70.1 ± 35.9 ^#1,2,3^	<0.001 **
HDL cholesterol (mg/dL)	55.9 ± 17.2 ^#2,3,4^	65.9 ± 16.8 ^#1,4^	64.3 ± 16.0 ^#1,4^	71.7 ± 16.7 ^#1,2,3^	<0.001 **
Serum uric Acid (mg/dL)	5.86 ± 1.45 ^#2,3,4^	4.92 ± 1.31 ^#1,4^	5.00 ± 1.31 ^#1,4^	4.45 ± 1.16 ^#1,2,3^	<0.001 **
Serum urea Nitrogen (mg/dL)	15.00 ± 4.13 ^#2^	16.39 ± 4.54 ^#1,3,4^	14.76 ± 3.97 ^#2^	13.96 ± 4.07 ^#12^	<0.001 **
Serum creatinin (mg/dL)	0.78 ± 0.21 ^#2,3,4^	0.69 ± 0.16 ^#1^	0.70 ± 0.15 ^#1,4^	0.66 ± 0.12 ^#1,2,3^	<0.001 **
uACR (mg/grCr)	62.3 ± 323.1	23.7 ± 50.2 ^#3,4^	20.7 ± 52.9 ^#2^	14.9 ± 19.1 ^#2^	0.008 **
Hypertension: n (%)	61 (59.2)	88 (64.7)	143 (45.4)	81 (24.3)	<0.001 **
Hyperlipidemia: n (%)	62 (60.2)	62 (45.6)	131 (41.7)	68 (20.4)	<0.001 **
Drinking alcohol: n (%)	50 (48.5)	53 (39.0)	137 (42.6)	142 (42.6)	0.521
Smoking (Never/Past/Current): n (%)	54/22/27 (52.4/26.2/21.4)	96/27/13 (70.6/19.9/9.6)	196/63/55 (62.4/20.1/17.5)	224/62/47 (67.3/18.6/14.1)	0.017 *

*p* < 0.05 and <0.01 are indicated by * and **, respectively. Data are mean ± SD or number of subjects (%). Statistical significance (*p* < 0.05) of differences in between-group parametric values assessed by Tukey’s post-hoc analysis after ANOVA is indicated by #, along with the cluster number, in which there was a significant difference.

**Table 3:** Risk of each cluster for incident diabetes.

	Univariate			Multiple Factors Adjusted		
	HR	95% CI	*p*	HR	95% CI	*p*
Clusters 3 + 4	Ref	-	-	Ref	-	-
Cluster 2	72.0	17.2–302.3	<0.001 **	53.2	12.4–227.5	<0.001 **
Cluster 1	25.8	5.5–121.6	<0.001 **	14.2	2.9–70.4	0.001 **

Multiple factors: age, gender, sBP, LDL-c, HDL-c, SUN, Cre, SUA, and alcohol drinking and smoking habits. *p* <0.01 is indicated by **.

**Table 4:** Changes in the clinical characteristics from baseline to diabetes onset in clusters 1 and 2.

	Cluster 1			Cluster 2		
Characteristics	Baseline	Onset	*p*	Baseline	Onset	*p*
Number (Men/Women)	8 (4/4)		-	28 (15/13)		-
Age (y)	60.0 ± 6.5	62.4 ± 7.0	<0.001 **	62.5 ± 12.2	65.0 ± 12.1	<0.001 **
Height (cm)	159.7 ± 9.5	159.6 ± 9.5	0.231	160.5 ± 10.1	160.3 ± 10.2	0.032 *
Body weight (kg)	69.81 ± 10.71	71.42 ± 11.06	0.071	62.35 ± 11.49	63.36 ± 12.61	0.019 *
Body mass index (kg/m^2^)	27.40 ± 3.72	28.06 ± 3.79	0.050 *	24.12 ± 3.33	24.58 ± 3.72	0.005 **
Fat (%)	31.84 ± 10.69	33.34 ± 10.51	0.078	25.18 ± 9.33	26.86 ± 9.27	<0.001 **
Plasma glucose (mg/dL)	88.88 ± 13.07	105.75 ± 13.48	0.005 **	97.61 ± 11.41	114.82 ± 17.32	<0.001 **
C-peptide (nmol/L)	2.01 ± 0.48	2.38 ± 1.17	0.365	1.13 ± 0.27	1.50 ± 0.58	0.003 **
HbA1c (%)	6.00 ± 0.22	6.25 ± 0.41	0.028 *	6.23 ± 0.16	6.54 ± 0.39	<0.001 **
HOMA2-IR	1.47 ± 0.39	1.84 ± 0.98	0.340	0.84 ± 0.20	1.18 ± 0.49	<0.001 **
HOMA2-B	129.9 ± 26.4	99.2 ± 20.3	0.029 *	72.9 ± 17.4	64.6 ± 18.8	0.005 **
Systolic blood pressure (mmHg)	139.8 ± 17.5	134.5 ± 16.9	0.429	145.2 ± 19.7	135.3 ± 15.7	0.010 **
Diastolic blood pressure (mmHg)	85.3 ± 8.3	80.5 ± 9.5	0.159	85.5 ± 11.2	83.7 ± 12.5	0.334
LDL cholesterol (mg/dL)	106.8 ± 43.2	113.6 ± 35.4	0.523	131.3 ± 28.5	124.2 ± 24.4	0.197
Triglyceride (mg/dL)	179.8 ± 115.2	195.3 ± 205.7	0.703	121.3 ± 99.9	121.4 ± 95.9	0.861
HDL cholesterol (mg/dL)	50.5 ± 9.67	52.3 ± 8.7	0.450	59.8 ± 17.8	60.5 ± 18.5	0.517
Serum uric Acid (mg/dL)	6.10 ± 1.58	6.60 ± 1.55	0.071	5.32 ± 1.28	5.41 ± 1.59	0.468
Serum urea Nitrogen (mg/dL)	14.28 ± 3.43	16.26 ± 3.67	0.053	16.08 ± 0.86	15.93 ± 3.71	0.785
Serum creatinin (mg/dL)	0.75 ± 0.14	0.78 ± 0.13	0.236	0.73 ± 0.15	0.73 ± 0.16	0.555
uACR (mg/grCr)	24.9 ± 34.1	25.0 ± 17.3	0.690	28.76 ± 58.28	18.1 ± 18.8	0.064
Hypertension: n (%)	7 (87.5)	8 (100.0)	0.317	22(78.6)	21(75.0)	0.564
Hyperlipidemia: n (%)	7 (87.5)	6 (75.0)	0.3179	13(46.4)	14(50.0)	0.655
Drinking alcohol: n (%)	3 (37.5)	3 (37.5)	>0.999	12(42.9)	12(42.9)	>0.999
Smoking (Never/Past/Current): n (%)	5/1/2 (62.5/12.5/25.0)	6/1/1 (75.0/12.5/12.5)	0.801	19/4/5 (67.9/14.3/17.9)	19/4/5 (67.9/14.3/17.9)	>0.999

*p* < 0.05 and <0.01 are indicated by * and **, respectively. Data are mean ± SD or number of subjects (%).

## Data Availability

All data generated or analyzed during this study are included in the published article.
